# Pristine Early Eocene Wood Buried Deeply in Kimberlite from Northern Canada

**DOI:** 10.1371/journal.pone.0045537

**Published:** 2012-09-19

**Authors:** Alexander P. Wolfe, Adam Z. Csank, Alberto V. Reyes, Ryan C. McKellar, Ralf Tappert, Karlis Muehlenbachs

**Affiliations:** 1 Department of Earth and Atmospheric Sciences, University of Alberta, Edmonton, Canada; 2 Environment and Natural Resources Institute, University of Alaska, Anchorage, Alaska, United States of America; 3 Department of Geoscience, University of Wisconsin, Madison, Wisconsin, United States of America; 4 Institute of Mineralogy and Petrography, University of Innsbruck, Innsbruck, Austria; The Pennsylvania State University, United States of America

## Abstract

We report exceptional preservation of fossil wood buried deeply in a kimberlite pipe that intruded northwestern Canada’s Slave Province 53.3±0.6 million years ago (Ma), revealed during excavation of diamond source rock. The wood originated from forest surrounding the eruption zone and collapsed into the diatreme before resettling in volcaniclastic kimberlite to depths >300 m, where it was mummified in a sterile environment. Anatomy of the unpermineralized wood permits conclusive identification to the genus *Metasequoia* (Cupressaceae). The wood yields genuine cellulose and occluded amber, both of which have been characterized spectroscopically and isotopically. From cellulose δ^18^O and δ^2^H measurements, we infer that Early Eocene paleoclimates in the western Canadian subarctic were 12–17°C warmer and four times wetter than present. Canadian kimberlites offer Lagerstätte-quality preservation of wood from a region with limited alternate sources of paleobotanical information.

## Introduction

**Figure 1:**
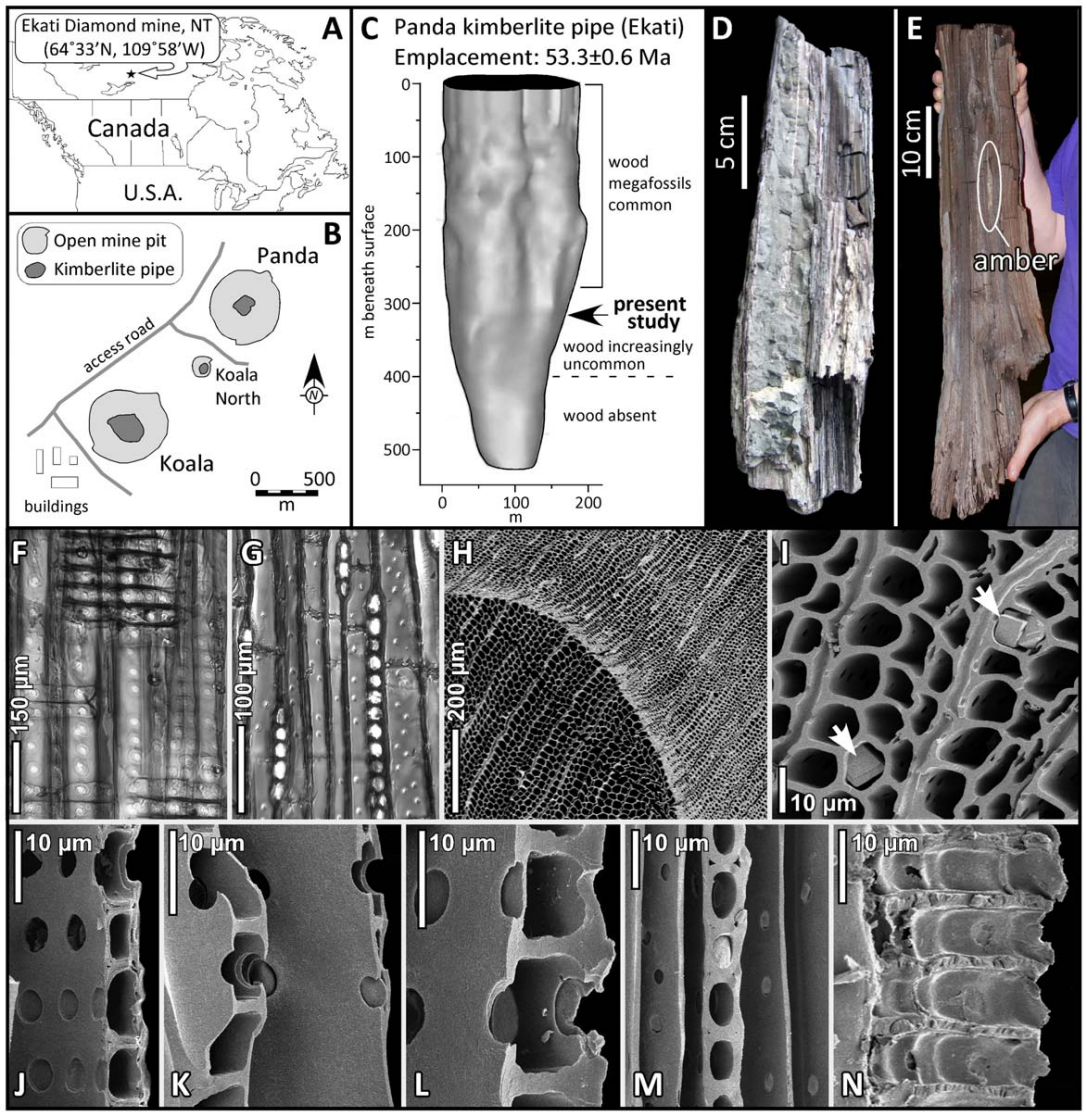
Study site and fossil wood from the Panda kimberlite pipe. A. Location of the Ekati diamond mine. B. Situation of the Panda kimberlite in relation to other pipes that comprise the property. C. Morphology of the Panda kimberlite pipe [8] and occurrence of wood. D. Fossil wood encrusted in olivine-rich volcaniclastic kimberlite. E. Photograph of the specimen characterized in this study. The wood was split when removed from the ore, revealing a sliver of opaque amber (9.5 cm long by 0.5 cm wide) in the xylem. F. RLS in transmitted light showing uniseriate and biseriate bordered pits and cross-fields. G. TLS showing rays stacked 3–26 cells high. H. SEM (TS) of ring boundary with earlywood (left) and latewood (right). I. Close-up of tracheids in TS and calcite crystals within cells (arrows). J. Cross-section of ray with cross-field pits. K and L. Close-ups of cross-field pits. M. TLS close-up of rays. N. Radial longitudinal section showing four contiguous rows of ray parenchyma cells with smooth end walls and no separation between the individual rows of cells.

Kimberlites are volatile-rich volcanic systems that ascend from the mantle episodically in Earth history as explosive phreatomagmatic events [1]. Fossils associated with kimberlites have been recognized for decades [2], [3]. Two modes of preservation are possible: (1) collapse or entrainment into the diatreme at the time of emplacement, resulting in the emtombment of fossiliferous xenoliths within the kimberlite body; and (2) accumulation in the crater following magmatism (kimberlite maar sedimentation). Both preservation types occur in kimberlite pipes of northwestern Canada’s Slave Province [4]–[7]. Although abundant wood has been recovered at depth from the Panda pipe (64.73°N, 110.59°W; Fig. 1) during mining of the diamondiferous ore body on BHP Billiton’s Ekati property, these remarkable botanical fossils have not yet been described in detail. We report here on the preservation and identification of unpermineralized wood recovered from the Panda kimberlite, and present an array of stable isotopic measurements that are used to derive a provisional reconstruction of regional paleoclimate during the Early Eocene.

The Panda kimberlite is one of approximately 150 pipes in the Lac de Gras field with emplacement ages ranging from 45 to 75 Ma [8]. The Panda pipe has a small diameter (∼200 m) and relatively simple geometry (Fig. 1), with no evidence of multiple phreatomagmatic events. Two additional pipes, Koala and Koala North, are emplaced immediately to the southwest of Panda; all three have been exploited by open-pit diamond mining. The age of the Panda intrusion has been dated precisely by Rb-Sr determinations on kimberlitic phlogopite (*n* = 7), yielding a robust isochron of 53.3±0.6 Ma (Early Eocene, Ypresian) [9]. Wood is common in the upper 300 m of the kimberlite body, and small rounded fragments (<10 cm) are often floated during rinsing of crushed ores. Larger wood fragments (>50 cm) have been retrieved directly from the ore before crushing; these are typically encrusted in reworked volcaniclastic kimberlite (Fig. 1D). We focus on one such specimen obtained from BHP Billiton and originating from the 315 m bench of the mine. The specimen is exceptional because of its size and the preservation of an amber nodule revealed within the xylem upon splitting (Fig. 1E). This offers the rare opportunity to conduct parallel geochemical investigations of the wood and associated amber, and to diagnose the wood anatomically. We envisage that the source tree collapsed into the diatreme at the time of kimberlite emplacement. The great depth of burial suggests that it entered a narrow marginal boundary layer between the blast zone and the wall rock before becoming entombed. We consider the wood to be representative of the Early Eocene forest growing at the site at the time of magmatism. The lack of permineralization suggests that burial was rapid, and that little post-eruptive thermal or tectonic alteration has occurred at the locality.

## Results and Discussion

### Wood Anatomy and Identity

Wood from the Panda kimberlite has pristine preservation (Fig. 1). Only the exterior of the specimen (<1 mm) is fusinized, implying that little free oxygen was present at the time of burial. Tracheids measure up to 62 µm in tangential diameter. In radial longitudinal section (RLS), earlywood tracheids exhibit uniseriate (58%) and biseriate (36%) bordered pits, with the remainder unpitted. Pits are circular (diameter: 15–20 µm) and are characterized by circular apertures (5–6 µm) arranged in contiguous chains of 1–8 cells. Multiseriate bordered pits have dominantly opposite arrangement (98%). Crassulae are present. Rays possess thin horizontal walls composed of cells up to 248 µm long, 10 µm high, and 12 µm wide. Cross-fields have 1–3 oppositely arranged taxodioid-cupressoid pits per field. Axial parenchyma consists of vertically stacked cells up to 160 µm in height, with both smooth (65%) and nodular (35%) end-walls. Axial tracheid walls are sparsely pitted in tangential longitudinal section (TLS). Rays are 3–26 cells high and dominantly uniseriate (97%). In transverse section (TS), growth rings are narrow (1.5–2.0 mm) with marked boundaries between latewood (35–49 µm) and earlywood (10–15 µm) cells. Near the ring boundary, rays are up to 2.5 mm long and spaced on average 70 µm apart (i.e. 12–14 rays per mm). Both horizontal and axial resin ducts are absent.

These features collectively identify the Panda wood as *Taxodioxylon* Hartig 1848 [10]–[12]. Moreover, fine anatomical details, including axial parenchyma with alternately nodular and smooth end-walls, single rows of taxodioid-cupressoid cross-field pits lacking separation between ray cells, and abrupt ring boundaries narrow the identification to *Metasequoia* Miki ex. Hu & Cheng 1948 [13]. *Metasequoia* was common in southern Alaska in the Late Paleocene and Early Eocene, producing a rich record of foliage and cones [14]. The genus is also abundant in younger (Middle Eocene, ca. 40 Ma) post-eruptive kimberlite maar sediments from the Slave Province, where it has been described as *M. occidentalis* (Newberry) Chaney [7]. The latter taxon is likely conspecific with the only extant congener, *M. glyptostroboides* Hu & Cheng, at present native only to isolated montane tracts in south-central China [13].

### Cellulose Preservation

**Figure 2:**
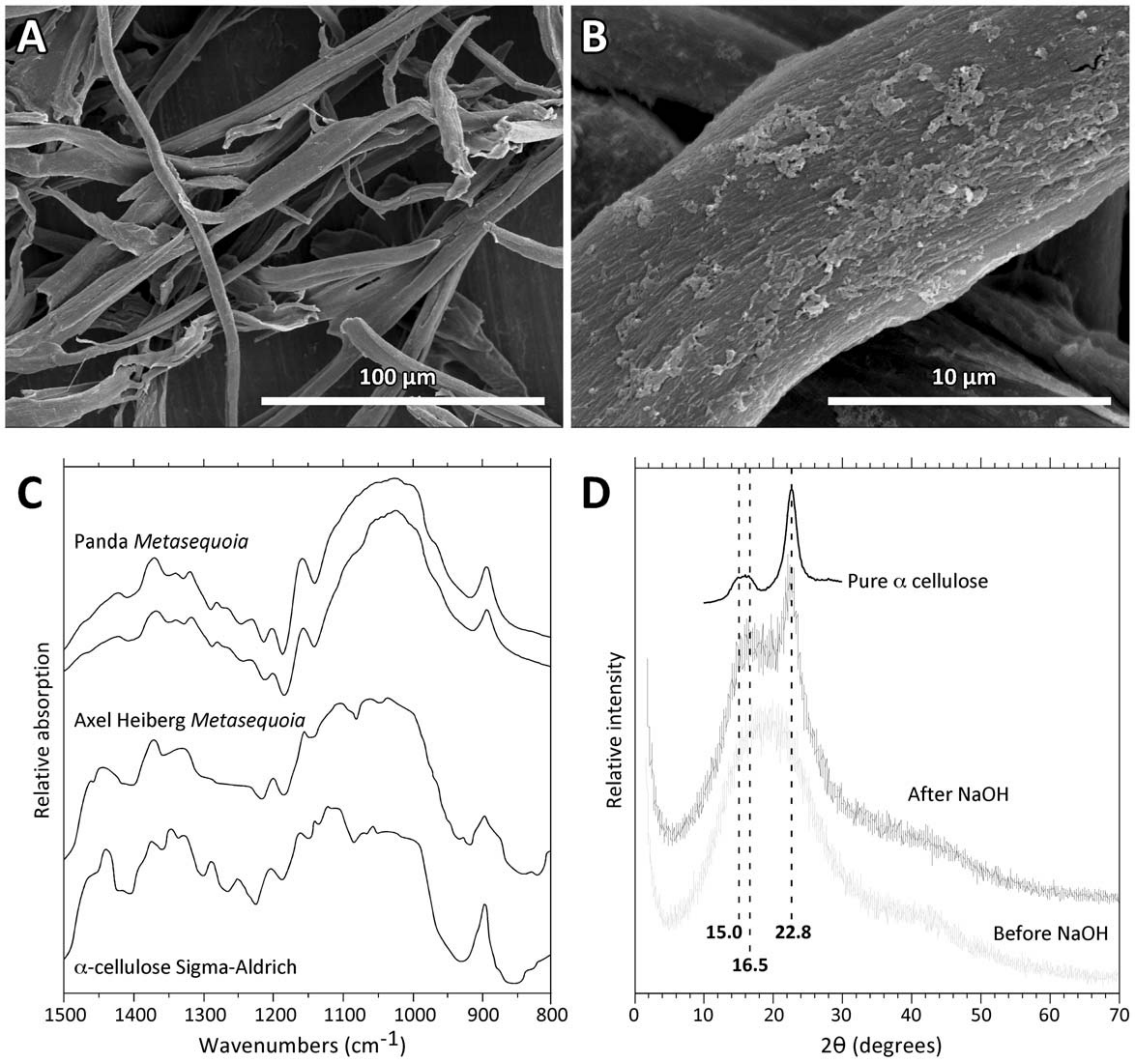
Cellulose extracted from Panda *Metasequoia*. A. SEM of cellulose fibers at low magnification. B. SEM at higher magnification showing surface texture. C. Duplicate FTIR spectra (800–1500 cm^−1^) of the same material, shown in relation to Middle Eocene cellulose from Axel Heiberg Island *Metasequoia* [15] and laboratory standard (Sigma-Aldrich) α-cellulose. D. X-ray diffraction traces of Panda cellulose extracts before (grey) and after (black) treatment with NaOH. Peaks associated with α-cellulose crystallinity are indicated by vertical dashed lines, whereas the idealized spectrum of pure α-cellulose is shown above the Panda traces [17].

Cellulose preservation in fossil conifers varies tremendously given the labile nature of constituent polysaccharides, mandating the need for quality control prior to isotopic analysis [15]. When viewed under scanning electron microscopy (SEM), extracts from Panda *Metasequoia* yield fibrous white material having the characteristic texture of wood cellulose (Fig. 2A–B). Fourier transform infrared (FTIR) spectra of the extracts support this contention: major absorption peaks including CH_2_ deformation (900 cm^−1^) and CH_3_ skeletal vibration (1375 cm^−1^) are common to both the Panda extracts and laboratory standard α-cellulose (Fig. 2C), yielding spectra that are fundamentally different from hemi- and holocellulose [16]. Furthermore, the removal of hemicellulose with NaOH results in markedly more crystalline x-ray diffraction patterns (Fig. 2D), which lends support to the contention that these extracts are primarily α-cellulose [17]. To our knowledge, this is the oldest verified instance of α-cellulose preservation to date, testifying to the remarkable preservation potential of kimberlite-hosted wood.

### Amber Spectroscopy and Thermal Alteration

**Figure 3:**
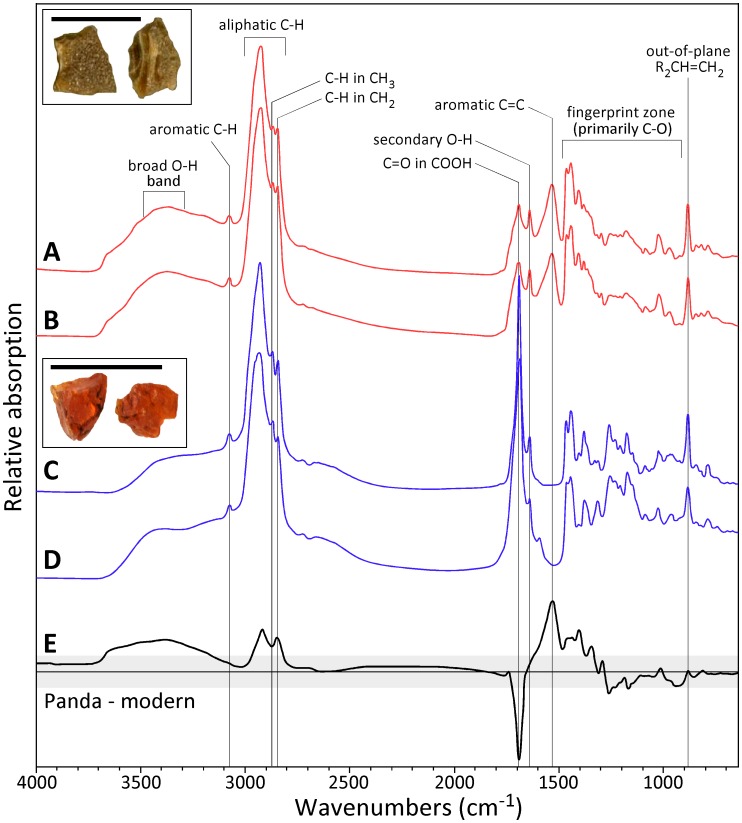
Complete FTIR spectra (650–4000 cm^−1^) of fossil and modern *Metasequoia* resins. A–B. duplicate analyses of the amber from Panda wood. C. Middle Eocene amber from post-eruptive sediments in the Giraffe kimberlite pipe, which has not been thermally altered [7], [19]. Insets show typical fragments of Panda and Giraffe *Metasequoia* ambers (scale bars are 5 mm). D. Modern resin from *M. glyptostroboides* cultivar (Washington DC, USA) is visually identical to the Giraffe material. E. The spectroscopic differences between Panda and modern *Metasequoia* resins reveals features associated with thermal maturation (grey area is±1 SD of the difference).

FTIR spectra of amber fragments from the Panda wood indicate that they are dominantly composed of polylabdanoid diterpenes in the absence of succinic acid; the material is therefore classified tentatively as a Class 1b amber, consistent with other deposits attributed to cupressaceous conifers [18]. Indeed, FTIR spectra conform to modern and unaltered fossil *Metasequoia* resins in several regards (Fig. 3). The out-of-plane C = H deformation bands typical of cupressaceous conifers (887 and 975 cm^−1^) are well expressed in both materials, as are the positions of peaks associated with C–O stretching (1030 cm^−1^), CH_2_ (2847 cm^−1^), and CH_3_ (2870 cm^−1^) [19]. However, the Panda specimens have stronger broad-band O–H (3300–3400 cm^−1^), markedly reduced C = O in COOH (1693 cm^−1^), and greatly enhanced aromatic C = C (1520 cm^−1^) absorption bands relative to other congeneric resins. These features are well expressed by the spectroscopic difference between Panda amber and modern *Metasequoia* resins (Fig. 3E), and are interpreted to reflect a diagenetic sequence involving decarboxylation of cyclic hydrocarbons to more aromatic compounds, as observed elsewhere in the thermal maturation of conifer resins [20]. Abiotic processes operating under anoxic conditions are responsible for these chemical transformations. Additional observations are consistent with some degree of thermal alteration in the Panda material, including the opacity of the amber owed to microscopic bubble inclusions (Fig. 3), the fusinization of wood outer surfaces, and the presence of calcite (CaCO_3_) crystals in wood cells (Fig. 1I), as confirmed by SEM-based energy dispersive spectroscopy. Calcite crystals are interpreted as *in situ* pseudomorphs following calcium oxalate (CaC_2_O_4_) initially produced by secondary metabolism in the living tree prior to burial [21].

Although it remains difficult to constrain eruptive temperatures during kimberlite emplacement due to the potential variability of volatile content and proximity to the local water table at the time of eruption, these are likely to have been in the 800–1200°C range initially, with the potential for considerable chilling (to 90–140°C) associated with adiabatic expansion during ascent [1], [22]. Thermal maturation of organic macerals from a range of Slave Province kimberlite diatreme facies has been evaluated by vitrinite reflectance, revealing maximum diagenetic temperatures of 350–450°C [23]. Wood from the Panda pipe has no cellular damage associated with devolatilization, whereas resin FTIR spectra provide no evidence for dehydration, confirming that the material was not exposed to exceedingly high temperatures (i.e. >500°C) upon burial. Despite the relatively subtle features attributed to thermal alteration noted above, we find little evidence that either the quality of cellulose preservation or the isotopic signatures of the various analyzed fractions have been overprinted. We thus envisage that cooling of the igneous body following emplacement in the diatreme was extremely rapid, potentially near-instantaneous [1], and surmise that any chemical changes to the entombed organic matter occurred in a closed system.

### Stable Isotopes and Paleoclimatic Inferences

Stable isotopic results from Panda cellulose and amber (Table 1) can be used to develop a range of inferences concerning the conditions under which tree growth occurred. Highly depleted mean δ^2^H values were obtained from nitrated wood cellulose samples (−160.5±3.8‰, *n* = 4). Using the accepted relationship between tree cellulose and surface water δ2H [24], this translates to an average δ2H of −138‰ for environmental waters accessed by the tree (Table 1). In the modern North American isoscape, this corresponds to a boreal environment [25]. In the Early Eocene, isotopically-depleted precipitation influenced drainage to the Arctic Ocean immediately following the Paleocene-Eocene Thermal Maximum (PETM; 55.5 Ma), inscribing low δ^2^H values on a range of terrestrial biomarkers [26]. Indeed, these authors propose a range of δ^2^H values for precipitation in the post-PETM Arctic (−105 to −145‰) that encompasses the inferred environmental water δ^2^H from Panda tree cellulose (−138‰).

**Table 1:** Stable isotopic ratios of amber and cellulose extracted from *Metasequoia* wood entombed in the Panda kimberlite.

Measurement	Minimum	Maximum	Mean	±1 s.d.	*n*
Amber δ^2^H (‰ VSMOW)	−371.09	−363.90	−368.95	2.59	6
Cellulose δ^2^H (‰ VSMOW)	−165.06	−156.34	−160.54	3.78	4
Inferred δ^2^H_environmental water_ (‰ VSMOW)^1^	−143.06	−134.34	−138.54	−	−
Amber δ^13^C (‰ VPDB)	−25.17	−24.56	−24.83	0.22	6
Cellulose δ^13^C (‰ VPDB)	−19.84	−19.65	−19.74	0.13	2
Cellulose δ^18^O (‰ VSMOW)	18.52	18.75	18.61	0.12	4
Inferred δ^18^O_environmental water_ (‰ VSMOW)^2^	−16.48	−16.25	−16.39	−	−

1 using ε_cellulose-environmental water_ = −22‰ [24].

2 using ε_cellulose-environmental water_ = 35‰ for *Metasequoia* [31].

Amber from the Panda locality also produced highly depleted δ^2^H values (−369.0±2.6‰, *n* = 6), although these are not exceptional by comparison to both Early Eocene cupressaceous ambers from Ellesmere Island (−354.2±5.3‰, *n* = 4; authors’ unpublished data) as well as mixed specimens from the Middle Eocene fossil forest of Axel Heiberg Island (−357.9±27.3‰, *n* = 6, [27]). Moreover, Panda amber δ^13^C values (−25.17±0.22‰) are well within the range reported for ambers of various age and provenance [28]. These similarities are inconsistent with the notion that the Panda amber isotopic signature has been altered during the burial process. Thus, the cellulose-based estimate of δ^2^H_environmental water_ derived above implies that Panda amber is depleted relative to water by 229‰ (range: 228–230‰). This fractionation is slightly less than the range (238–303‰) obtained experimentally for diterpenoids synthesized by the non-mevalonate (or MEP/DOXP: 2-*C*-methyl-D-erythritol 4-phosphate/1-deoxy-D-xylulose 5-phosphate) pathway in the living cupressaceous conifer *Cryptomeria japonica* [29]. As this biochemical pathway is an essential precursor to the resin diterpenoids that polymerize to form amber, we consider the Panda δ^2^H values to capture the primary isotopic signature of resin metabolism, with no discernible evidence of diagenetic overprinting. Recent experimental observations, where *Metasequoia* resins were heated at 90°C in deuterated water and sampled monthly over a period of one year, support the notion of limited exchangeability of H (i.e. <5%) following initial resin biosynthesis [30].

The δ^18^O of Panda cellulose ranges from 18.5–18.8‰, implying a mean δ^18^O_environmental water_ of −16.4‰ using a constant enrichment of 35‰ between cellulose and water observed in modern *Metasequoia* [31]. Using schemes based on cellulose δ^13^C [32] and paired δ^18^O and δ^2^H [33], we computed a likely range of ambient relative humidity (RH) values, resulting in mean, minimum, and maximum RH of 77%, 64% and 83%, respectively. These estimates are consistent with the RH of 75% derived independently from an Early Eocene (52–53 Ma) vertebrate assemblage on Ellesmere Island [34]. We then applied these RH estimates to estimate δ^18^O_precipitation_ using two alternate methods [35], [36] and derived corresponding mean annual temperatures using Eocene latitudinal gradients [37]. These results produce MAT estimates of 7–12°C, with a grand mean of 9°C (Table 2). Present MAT at Yellowknife is −4.6°C [38]. Thus, in the Early Eocene, the Panda locality was in the order of 12–17°C warmer than present.

**Table 2:** Two estimates of Early Eocene δ^18^O_precipitation_ [35], [36] and corresponding mean annual temperature (MAT) [37] derived from Panda cellulose for minimum, mean, and maximum inferred values of relative humidity (RH).

Relative humidity		ref. [26]	ref. [27]
RH = 64%			
	δ^18^O_precipitation_ (‰)	−13.5	−12.9
	MAT (°C)	10.5	7.0
RH = 77%			
	δ^18^O_precipitation_ (‰)	−15.9	−14.0
	MAT (°C)	7.7	10.3
RH = 83%			
	δ^18^O_precipitation_ (‰)	−17.0	−17.4
	MAT (°C)	6.4	12.0

These results can be compared to the MAT of 8°C and warmest month temperature of 19–20°C inferred for the Early Eocene on Ellesmere Island [34]. Leaf margin analyses from Paleocene-Eocene sediments in southern Alaska produce MAT estimates of 11–14°C and warmest month temperatures of 22°C [14]. Together, these estimates converge on the warm-temperate character of Early Eocene high-latitude climates, with greatly reduced seasonality relative to present. At the same time, conditions at the Panda locality must have been considerably more humid than present. This is because *Metasequoia* requires >1000 mm of annual precipitation in order to thrive [39], roughly four times more precipitation than received at Yellowknife today (280 mm, [38]). The humid conditions envisaged for the Arctic Ocean basin during the Early Eocene [40] clearly extended to subarctic latitudes of the North American interior.

**Figure 4:**
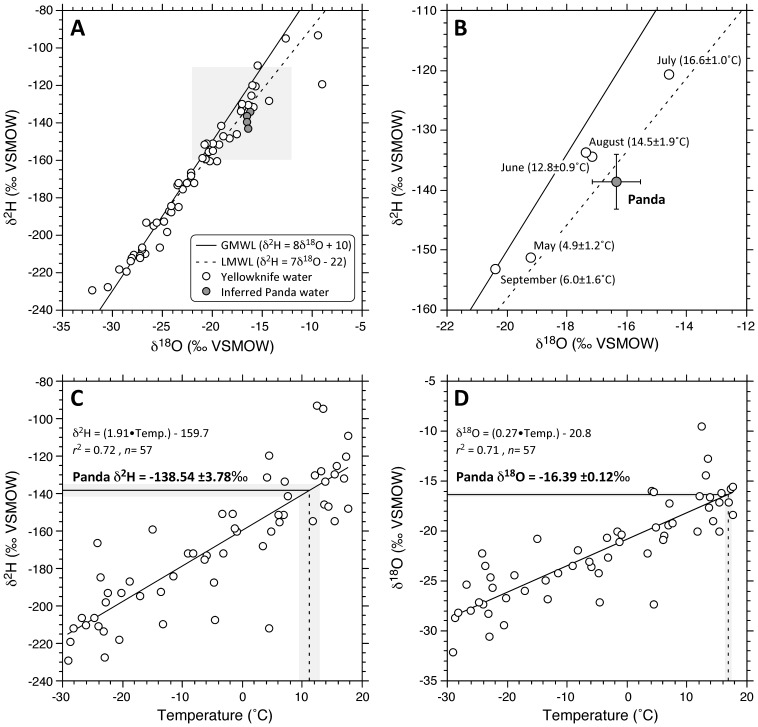
Mean monthly δ^18^O and δ^2^H of precipitation at Yellowknife (Station 7193401, 62.28°N, 114.27°W, 205 m, [41]), shown in relation to inferred values of environmental waters accessed by Panda *Metasequoia*. A. Monthly values for the five-year measurement period (1989–1993) depict a local meteoric water line (LMWL) that deviates only slightly from the Global Meteoric Water Line (GMWL). The Panda cellulose isotopic values, once converted to the composition of environmental water (Table 1), plot on the LMWL. B. Magnification of the shaded grey in A, showing mean monthly isotopic values for months between May and September, corresponding mean monthly temperatures for the observation period (*n* = 5), and the mean value derived from Panda wood cellulose (*n* = 4). C. Temperature dependency of modern precipitation δ^2^H at Yellowknife and the inferred range of values obtained from Panda cellulose, with extrapolated temperature estimates (dashed vertical line). D. As for C, but for precipitation δ^18^O.

The MAT reconstructions presented in Table 2 are corroborated by an additional analysis: the comparison of Panda cellulose isotopic composition with that of precipitation at Yellowknife, the nearest station (314 km to the southwest) for which a multi-year characterization exists for both δ^2^H and δ^18^O [41]. The δ^2^H_environmental water_ and δ^18^O_environmental water_ values obtained from Panda wood cellulose using constant enrichment factors between water and cellulose were used (Table 1), in order to minimize the effects of assumptions regarding RH. It can be shown that the δ^2^H and δ^18^O of source waters inferred from Panda wood cellulose plot closely to the local meteoric water line (Fig. 4A), strongly supporting the contention that mechanisms of Early Eocene atmospheric water transport bore remarkable similarities to the modern world [15], despite pronounced differences in precipitation quantity, ambient temperature, and seasonality. Moreoever, the Panda data plot intermediately between the mean modern Yellowknife values for May and September, and those for June, July and August, the warmest months (Fig. 4B). The corresponding monthly mean temperatures are entirely compatible with MAT estimates based on empirical relationships applied to the Panda wood isotopic composition (Table 2). When the modern temperature dependencies of precipitation δ^2^H and δ^18^O from Yellowknife are plotted independently, inferred environmental water isotopic values from Panda cellulose imply temperatures of 11°C and 17°C, respectively (Fig. 4C–D). These values are several degrees warmer than the MAT estimates of Table 2, and are thus likely to reflect the warm season of active biomass accrual only. From these various analyses, we surmise that the Panda cellulose isotopic measurements provide robust and highly coherent paleoclimatic information for the Early Eocene in northwestern Canada, thus filling a large geographic void with respect to the closest contemporaneous fossil localities [14], [34].

### Conclusions

Due to the coupled effects of multiple denudational phases and pervasive Neogene glacial erosion, the ∼210,000 km^2^ expanse of the Slave Craton lacks traditional Phanerozoic fossil exposures [42]. Kimberlites revealed by diamond exploration and mining thus provide the sole repository of paleobiological information for this large expanse of Northwestern Canada. Although deep burial in volcaniclastic kimberlite is an unconventional source for Lagerstätte-quality plant megafossils, we have demonstrated that such a record indeed exists in the Panda pipe, and likely in other Slave Province kimberlitic intrusions where wood has been documented [4], [8], [23]. The state of preservation of this wood is unequalled for material of this age, as exemplified by the exquisite quality of anatomical detail and the presence of α-cellulose. This exceptional preservation has enabled a range of isotopic measurements that provide a provisional reconstruction of the mean climate state under which tree growth occurred. In the Early Eocene, immediately following peak Cenozoic warmth driven by enhanced greenhouse gas forcing [43], the subarctic latitudes of the Slave Province harbored *Metasequoia* in forests developed under conditions 12–17°C warmer and four times wetter than at present.

## Materials and Methods

The wood specimen considered here was archived in the core lab of BHP Billiton Diamonds Inc., then located in Kelowna, British Columbia, Canada. BHP granted permission to sample the Panda wood material, and donated the specimen to the University of Alberta for further analyses. No additional permissions were required to undertake the study.

We sectioned transverse (TS), radial longitudinal (RLS) and tangential longitudinal (TLS) planes of the wood, embedded them under vacuum in Epothin, and ground thin sections for examination under transmitted light microscopy. The wood was easily dissected by scalpel. The same planes of unimpregnated wood were also examined using field-emission SEM on a JEOL-6301F system operating at 25 kV, following sputter-coating with Au. For isotopic analyses, cellulose was extracted from wood samples using a modified Jayme-Wise method [44], where ground samples were refluxed by soxhlet in toluene and ethanol to remove resins, delignified in an acidified NaClO_2_ solution, washed in NaOH to remove hemicellulose, and leached of Fe and Mn oxyhydroxides using hydroxylamine hydrochloride (H_3_NO•HCl). Purified cellulose extracts were examined by SEM and characterized by FTIR spectroscopy with a Thermo-Nicolet Nexus 470 bench spectrometer fitted with a dual-aperture Continuum infrared microscope. These extracts were also analyzed by x-ray diffraction, using a Rigaku Geigerflex powder diffractometer equipped with a cobalt tube, graphite monochromator, and scintillation detector. The occluded amber was also analyzed by FTIR and compared to a broad array of modern and fossil resin spectra [19].

A range of stable isotopic measurements were made on these materials: δ^2^H (*n* = 4), δ^13^C (*n* = 2) and δ^18^O (*n* = 4) were measured from wood cellulose extracts, whereas δ^2^H (*n* = 6) and δ^13^C (*n* = 6) were measured from untreated amber fragments. Prior to the measurement of δ^2^H, cellulose was nitrated with phosphorus pentoxide to remove exchangeable H [45]. Cellulose δ^2^H and δ^18^O were measured at the University of Arizona using a Thermo Finnigan Delta Plus XL isotope-ratio mass spectrometer in continuous flow mode coupled to a Thermo Finnigan TC/EA via a Conflo III interface. Carbon and hydrogen isotope ratios of amber, and carbon isotopes of cellulose, were measured at the University of Alberta using a Finnigan MAT-252 isotope-ratio mass spectrometer in dual-inlet mode. Isotopic results are expressed in δ notation relative to Vienna Standard Mean Ocean Water (VSMOW) for δD and δ^18^O, and Pee Dee Belemnite (VPDB) for δ^13^C. Precision was±3‰ for δD and±0.1‰ for δ^13^C and δ^18^O.
